# Expression of *Trypanosoma brucei gambiense* Antigens in *Leishmania tarentolae*. Potential for Use in Rapid Serodiagnostic Tests (RDTs)

**DOI:** 10.1371/journal.pntd.0004271

**Published:** 2015-12-09

**Authors:** Barrie Rooney, Turid Piening, Philippe Büscher, Stijn Rogé, C. Mark Smales

**Affiliations:** 1 Centre for Molecular Processing, School of Biosciences, University of Kent, Canterbury, Kent, United Kingdom; 2 Medecins sans Frontieres, Amsterdam, The Netherlands; 3 Unit of Parasite Diagnostics, Department of Biomedical Sciences, Institute of Tropical Medicine, Antwerpen, Belgium; McGill University, CANADA

## Abstract

The development of rapid serodiagnostic tests for sleeping sickness and other diseases caused by kinetoplastids relies on the affordable production of parasite-specific recombinant antigens. Here, we describe the production of recombinant antigens from *Trypanosoma brucei gambiense* (*T*.*b*. *gambiense*) in the related species *Leishmania tarentolae (L*. *tarentolae)*, and compare their diagnostic sensitivity and specificity to native antigens currently used in diagnostic kits against a panel of human sera. A number of *T*.*b*. *gambiense* protein antigen candidates were chosen for recombinant expression in *L*. *tarentolae* based on current diagnostics in field use and recent findings on immunodiagnostic antigens found by proteomic profiling. In particular, the extracellular domains of invariant surface glycoprotein 65 (ISG65), variant surface glycoproteins VSG LiTat 1.3 and VSG LiTat 1.5 were fused with C-terminal histidine tags and expressed as soluble proteins in the medium of cultured, recombinant *L*. *tarentolae*. Using affinity chromatography, on average 10 mg/L of recombinant protein was purified from cultures and subsequently tested against a panel of sera from sleeping sickness patients from controls, i.e. persons without sleeping sickness living in HAT endemic countries. The evaluation on sera from 172 *T*.*b*. *gambiense* human African trypanosomiasis (HAT) patients and from 119 controls showed very high diagnostic potential of the two recombinant VSG and the rISG65 fragments with areas under the curve between 0.97 and 0.98 compared to 0.98 and 0.99 with native VSG LiTat 1.3 and VSG LiTat 1.5 (statistically not different). Evaluation on sera from 78 *T*.*b*. *rhodesiense* HAT patients and from 100 controls showed an acceptable diagnostic potential of rISG65 with an area under the curve of 0.83. These results indicate that a combination of these recombinant antigens has the potential to be used in next generation rapid serodiagnostic tests. In addition, the *L*. *tarentolae* expression system enables simple, cheap and efficient production of recombinant kinetoplatid proteins for use in diagnostic, vaccine and drug discovery research that does not rely on animal use to generate materials.

## Author Summary

The development of rapid serodiagnostic tests for African sleeping sickness and other diseases caused by kinetoplastids relies in part on the affordable production of parasite-specific recombinant antigens. The majority of cases of sleeping sickness are caused by the parasite *Trypanosoma brucei gambiense* (*T*.*b*. *gambiense*) which is transmitted when bitten by an infected tsetse fly. Existing tests rely on the utilisation of extracts from the parasite or use antigens raised in animal models. In this study we have shown that using a cell culture system devised from a parasite similar to *T*.*b*. *gambiense* recombinant antigens can be produced that are as effective in rapid diagnostic tests as the native antigens purified from *T*.*b*. *gambiense* parasites grown in laboratory rodents. We compared the diagnostic sensitivity and specificity of the antigens we produced recombinantly to native antigens currently used in diagnostic kits against a panel of human sera. The evaluation on sera from 172 *T*.*b*. *gambiense* patients and from 119 controls without sleeping sickness showed very high diagnostic potential of two recombinant antigens where the response was not significantly different to that from the native antigens. These results indicate that a combination of these recombinant antigens has the potential to be used in next generation rapid serodiagnostic tests.

## Introduction

Human African Trypanosomiasis (HAT), also known as African sleeping sickness, is usually a fatal disease caused by the parasites *Trypanosoma brucei gambiense* (*T*.*b*. *gambiense*) or *T*.*b*. *rhodesiense* [1–3]. The parasite is transmitted by the bite of infected tsetse flies in sub-Saharan Africa with *T*.*b*. *gambiense* being responsible for 95% of the cases in West and Central Africa. The remaining infections are caused by *T*.*b*. *rhodesiense* in East and Southern Africa. Although the number of infections currently reported at less than 5,000 cases per year are not at the level reported during the last century (300,000 per year), this disease still causes considerable suffering and burden on communities in terms of disability-adjusted life years [4,5]. The disease follows two stages where the trypanosomes are limited to the blood and lymphatic systems initially, but in most cases will invade the central nervous system. This second stage causes sleep cycle disruption and neurological damage leading to coma and death if not treated [6].

Diagnosis of sleeping sickness is complex as detection of the parasite in a patient is very difficult and, in the case of a positive result, involves a subsequent lumbar puncture to assess the disease stage [7]. Following an infective bite, the patient suffers peaks of parasitaemia as the host mounts a humoral response to the changing surface antigens on the trypanosome, in particular the variant surface glycoproteins (VSGs). Parasites attempt to evade this humoral attack by expressing alternative VSGs (antigenic variation) and continue to multiply until the new variable antigen type (VAT) is recognised by the host and attacked [8]. One of the most common VAT is considered to be LiTat 1.3 and its VSG is the major antigen present in the Card Agglutination Test for Trypanosomiasis (CATT) which is currently used in large scale population screening [9]. In the field, this is followed by a series of time-consuming parasitological tests that often are used to reach a diagnosis by the application of a complex algorithm [10].

Recent efforts to improve diagnosis have focussed on developing rapid immunochromatography-based serodiagnostic tests (RDT’s), which as point of care tests (POCT), should follow the WHO ASSURED criteria; affordable, sensitive, specific, user-friendly, rapid, equipment-free and deliverable to the people at need [11]. First generation RDTs look very promising but have lower specificity than expected when tested on a wider target area [12–14]. Current RDTs for HAT rely on the infection of rodents and the purification of the native VSGs LiTat 1.3 and LiTat 1.5, which do not represent all the variants encountered in the field. Other approaches to identifying immunodiagnostic antigens have involved soluble fractions of other native VSGs (sVSG117) and the proteomic identification and selection of other surface antigens [15,16]. The latter has identified a number of antigens that were recognised by the sera of HAT infected patients and in particular the invariant surface glycoprotein 65 (ISG65) showed potential, either on its own or in combination with sVSG117. Other potential antigens could not be expressed as recombinant proteins in *E*.*coli* and were not investigated further. However, unlike prokaryotic expression systems, eukaryotes have the ability to carry out more complex folding and post-translational modifications (PTMs) such as glycosylation and *Pichia pastoris* has recently been used successfully to express soluble VSGs [17]. Sugar moieties are known to be major antigenic structures by themselves and /or in combination with their folded protein partner [18]. For recombinant expression the main aim is to reproducibly produce material as close to the native type as possible without having to handle highly virulent parasites, and in an economic system. In this study we have examined the potential application of the related trypanosomatid organism *Leishmania tarentolae (L*. *tarentolae)* as a recombinant antigen production system [19]. It is a Biosafety handling level one system, which can be genetically engineered and scaled up to bioreactor production using readily available medium and equipment. We have expressed, purified and compared recombinant VSG fragments from *L*. *tarentolae*, with VSG’s purified from their native counterparts. We have also examined the potential of rISG65 from *L*. *tarentolae* to act as an antigen in serodiagnostic tests for sleeping sickness caused by both *T*.*b*. *gambiense* and *T*.*b*. *rhodesiense*. Testing was carried out on serum from confirmed HAT patients and controls from a wide range of endemic areas. We show that the *L*. *tarentolae* system can express recombinant antigens that are as effective as the native antigens for use in RDTs for HAT, and suggest this may have a wider application as a production platform of recombinant proteins for diagnostic and vaccine generation applications.

## Materials and Methods

### Ethics statement

Part of the serum samples used in this study were collected within a diagnostic study (SeroStrip) carried out in the Democratic Republic of the Congo (DRC) [13]. Permission for this study was obtained from the national ethics committee of DRC (CNG/M.D./111/2012) and from the ethics committee of the University of Antwerp (11435795). The other sera were obtained from the World Health Organization HAT Specimen Bank [20]. The specimen collection and banking was approved by the WHO Ethical Review Committee and the different national ethical committees in each country where specimens were collected. The National Ministries of Health also gave their approval. All individuals gave their written informed consent for the use of their plasma specimen in HAT research before providing blood. All specimens were anonymised.

### Specimen collection

Table 1 represents the serum collection used in this study. Serum donors were classified as HAT patients when trypanosomes were detected in any body fluid (blood, lymph, cerebrospinal fluid). Non-HAT controls were persons from HAT endemic regions but without history of HAT and without clinical, serological or parasitological evidence of infection with *T*.*b*. *gambiense* or *T*.*b*. *rhodesiense*. One hundred forty one sera from *gambiense* HAT (*g-*HAT) patients and from non-*g*-HAT controls were collected within the SeroStrip study conducted in the DRC [13]. Additional sera from *g-*HAT patients and non-*g*-HAT controls and from *rhodesiense* HAT (*r-*HAT) patients and non-*r*-HAT controls were received from the World Health Organization HAT Specimen Bank [20] and originated from Guinea, Tchad and DRC (*T*.*b*. *gambiense*) and from Malawi and Tanzania (*T*.*b*. *rhodesiense*).

**Table 1:** Serum collection used in this study.

	*T*.*b*. *gambiense*		*T*.*b*. *rhodesiense*	
	*g*-HAT patient	non-*g*-HAT control	*r-*HAT patient	non-*r*-HAT control
RDC	118	103		
Guinea	20	8		
Tchad	34	8		
Malawi			69	25
Tanzania			9	25

### Constructs and expression

Peptides were identified using data from proteomic studies on proteins recognised by *g*-HAT patient sera that were matched to an ISG65 from *T*.*b*. *brucei* (UniProt reference Q26712 and Q58F5) [16]. Using protein-protein BLAST a 98% identical *T*.*b*. *gambiense* homologue (UniProt reference C9ZJ77) was selected for expression and investigation in this project. The DNA coding for amino acid residues 19–385 of this homologue was commercially synthesized by Gene Art (LifeTechnologies). The DNA for recombinant VSG LiTat 1.3 (UniProt reference X5GEX5) and VSG LiTat 1.5 (UniProt reference E7EDN2) were kindly donated by S. Rogé, ITM, Belgium [17]. It had previously been shown that N-terminal peptides contain more specific epitopes [17] so the native DNA sequences coding for amino acid residues 24–372 for VSG LiTat1.3 and amino acids 33–426 for VSG LiTat 1.5 were used. All constructs were cloned into the LEXSYS vector pLEX hyg2 (Jena Bioscience) in frame with the signal peptide of secreted acid phosphatase of *L*.*mexicana* and a carboxy-terminal hexa-histidine tag within the vector. Following electroporation of LEXSYS host strain p10, clonal isolates were selected as previously described [19]. Production of recombinant protein was carried out in 1 litre baffled Erlenmeyer flasks in BHI medium (supplemented with antibiotics and hemin) and the medium was harvested when the OD_600_ reached 4 (approx. 70 h post inoculation, 10^8^ cells/ml). All media components were from Jena Bioscience. Clarified medium was concentrated twenty fold on a Pellicon XL 50 Ultrafiltration cassette (10 kDa MWCO) and diluted four times in binding buffer (20 mM Phosphate, 500 mM NaCl, 10 mM Imidazole) before addition to an equilibrated His Trap (GE Healthcare) FPLC column. Bound proteins were eluted using an increasing Imidazole gradient (20 mM Phosphate, 500 mM NaCl, 500 mM Imidazole). Peak protein containing fractions, as determined by A280 nm measurement, were combined, desalted and concentrated by centrifugation in Amicon Ultra-15 device (30 kDa MWCO). The final protein was stored at 1 mg/ml in solution in PBS, 15% glycerol at -20°C.

### Characterisation of recombinant proteins

**Fig 1:**
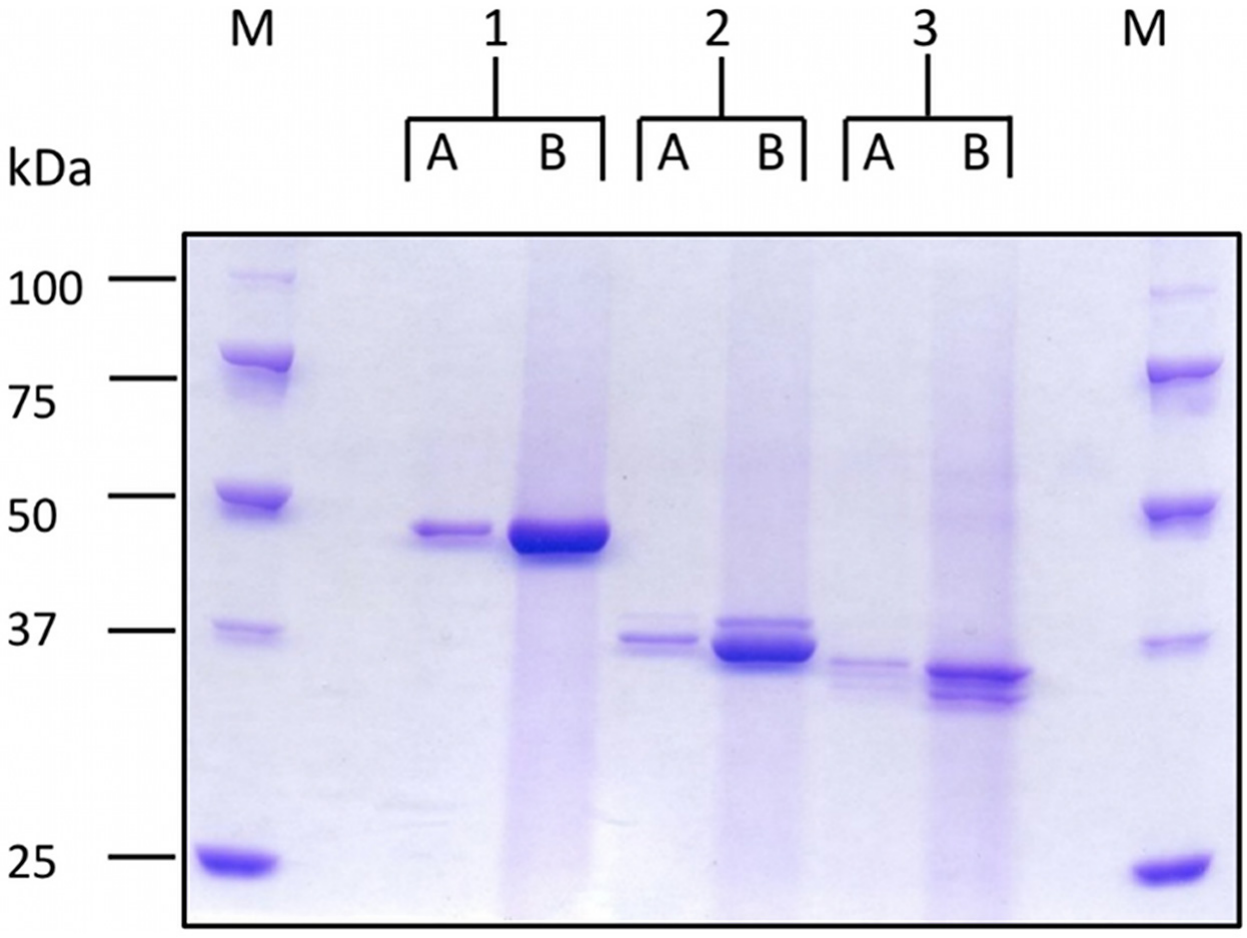
SDS-PAGE analysis of the expression of rISG65, rLiTat1.3 and rLiTat1.5 by *Leishmania tarentolae* after purification following a single round of metal affinity chromatography. Coomassie stained 10% SDS-PAGE; Precision Plus Protein Unstained Standards, BioRad (M). Lane 1: rISG65, Lane 2: rLiTat1.3, Lane 3: rLiTat1.5. A = 1 μg and B = 10 μg protein.

Following SDS-PAGE of purified recombinant proteins (Fig 1) each band was excised from the gel, digested with Trypsin (Sigma–Aldrich, P7367) and the resulting peptides subjected *to* matrix-assisted laser desorption ionization time of flight mass spectrometry *(*MALDI-TOF-MS. Briefly, excised bands were reduced and alkylated with iodoacetamide and digested in-gel with trypsin (Promega V5111) as previously described [21]. The tryptic peptides (0.5 μl) were spotted onto an MTP AnchorChip 384 T F target plate (Bruker part number 209514) and allowed to air dry. Peptides spots were then overlaid with an equal volume of α-cyano-4-hydroxycinnamic acid matrix (0.7 mg/ml in 85% acetonitrile, 15% water, 0.1% TFA and 1 mM NH_4_H_2_PO_4_, Bruker). The same volume of peptide calibration standard II mix (Bruker, made according to the manufacturer’s instructions) was spotted in the appropriate positions and allowed to air dry. Mass spectra were collected on an UltrafleXtreme (Bruker) instrument and Mascot software used to match peptides to the SwissProt database.

For gel filtration experiments, purified rISG 65 (100 μl of 1 mg/ml) was applied to a gel filtration Superdex 200 10/300 GL column (GE Healthcare LifeSciences) in sodium phosphate buffer (pH7.5), 0.5 M NaCl previously calibrated with molecular weight markers (Sigma-Aldrich MWGF100) and eluted with at a flow rate of 1 ml/min.

In order to examine the potential presence of N-linked sugars, rISG 65 (1 μg) was treated with PNGase F (1.5 U) (Sigma-Aldrich, P7367) for two hours at 37°C using manufacturers instructions before being analysed by SDS-PAGE to determine if this resulted in a shift in molecular weight.

### Native antigens

Native variant surface glycoprotein (VSG) was prepared following standard procedures from cloned populations of *T*.*b*. *gambiense* variant antigen types (VATs) LiTat 1.3 and LiTat 1.5 [22]. After purification, the native VSG LiTat 1.3 (nLiTat 1.3) and native VSG LiTat 1.5 (nLiTat 1.5) antigens were lyophilised in aliquots of 1 mg and stored at -80°C prior to use.

### ELISA

The ELISA protocol was based on the procedure according to Lejon *et al*. [23–25]. Microplates (Maxisorp, Nunc) were coated overnight at 4°C with 100 μl/well of purified recombinant protein at 4 μg/ml or with native antigen at 2 μg/ml. All antigens were diluted in phosphate buffer (PB) (10 mM sodium phosphate, pH 6.5). To correct for aspecific reactions, caused by contaminating *L*. *tarentolae* proteins that were not eliminated from the protein mixture by the one-step affinity purification, control wells were coated with the supernatant of a culture of untransfected *L*. *tarentolae* cells at 4 μg/ml. Further manipulations were undertaken at ambient temperature. After coating, the wells were blocked with PBS-Blotto (0.01 M sodium phosphate, 0.2 M sodium chloride, 0.05% NaN_3_, 1% skimmed milk powder, pH 7.4) for 1 hour. Before addition to the microplate the sera were diluted at 1:150 in PBS-Blotto. Antibody binding was visualised with goat anti-human IgG (H+L) conjugated with horseradish peroxidase (1:40000; Jackson ImmunoResearch) and the chromogen ABTS (2,2’-azinobis[3-ethylbenzothiazonline-6-sulfonic acid]-diammonium salt; Roche). The optical densities (ODs) were read at 414 nm (Multiskan RC Version 6.0; Labsystems). Corrected optical density (OD_corr_) values were calculated by subtracting for each serum the OD reading in the control well from the OD reading in the antigen coated well.

### Statistical analysis

ELISA results were captured in a Microsoft Excel 2010 database. The accuracy of the different antigens for diagnosis was determined in SigmaPlot 12.5 by calculation of the area under the receiver operator characteristics (ROC) curve (AUC) [26]. Confidence intervals (CI) were determined according to DeLong [27]. Sensitivities and specificities with 95% binomial Wilson confidence intervals and the Youden index were calculated using SigmaPlot 12.5 [28]. The McNemar Chi^2^ test was used to test differences in the AUCs.

## Results

### Production of recombinant antigens

**Fig 2:**
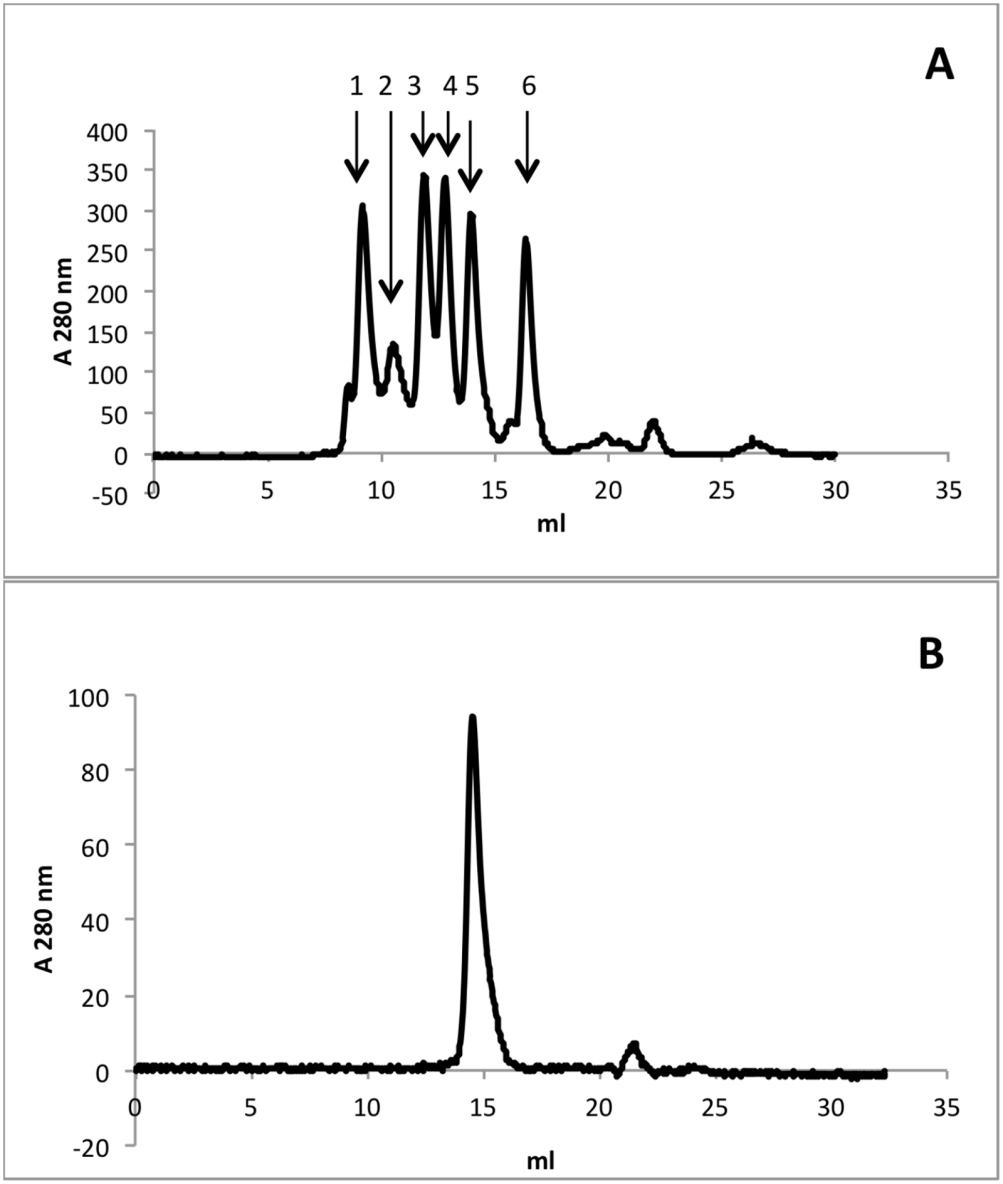
Gel filtration of purified rISG65 on a Superdex 200 10/300 GL column (GE Healthcare Lifesciences) in sodium phosphate buffer, pH7.5, O.5 M NaCl. A. Molecular weight markers (Sigma-Aldrich MWGF100) 1 = thyroglobulin 669 kDa, 2 = apoferritin 443 kDa, 3 = β-amylase 200 kDa, 4 = alcohol dehydrogenase 150 kDa, 5 = bovine serum albumin 66 kDa, 6 = carbonic anhydrase 29 kDa. B. Elution profile of rISG following one round of metal affinity chromatography purification.

**Fig 3:**
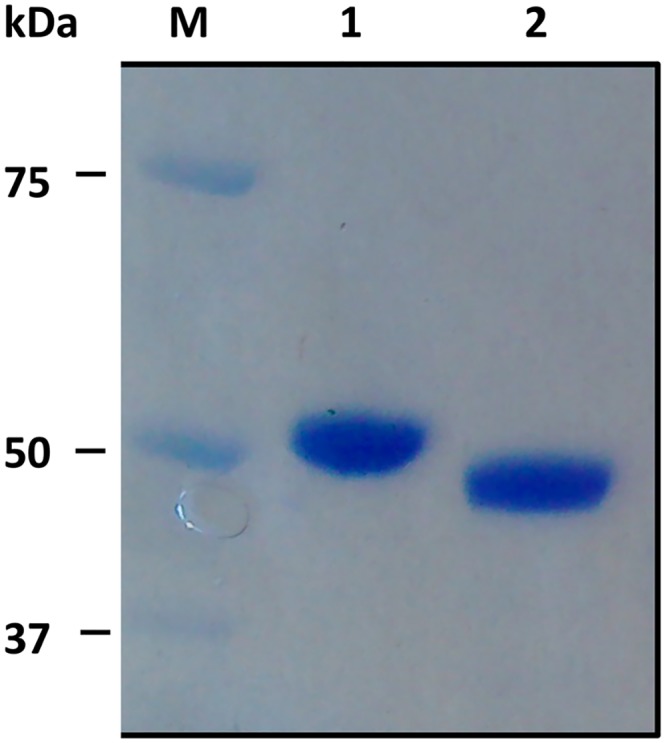
Analysis of the deglycosylation of rISG65 using PNGase F (1.5 U) (Sigma-Aldrich, P7367) for two hours at 37°C. Coomassie blue stained pre-cast 4–12% BisTris gradient SDS-PAGE gel (Novex) using the MOPS running system; Precision Plus Protein Unstained Standards, BioRad (M). Lane 1: rISG65 (1 ug) before treatment with PNGase F, Lane 2: rISG65 (1 ug) after treatment with PNGase F.

Soluble forms of the *T*.*b*. *gambiense* invariant surface glycoprotein 65 (ISG65) and the variant surface glycoproteins VSG LiTat 1.3 and VSG LiTat 1.5 (with C-terminal histidine tags remaining intact) were expressed as soluble proteins in the medium of the recombinant LEXSYS host strain p10 and purified on Ni-NTA resin. Protein purity was examined by SDS-PAGE analysis and subsequent Coomassie blue staining of the resulting gel to visualise those protein bands present (Fig 1). The identity of the recombinant proteins were confirmed by tryptic digestion of the excised bands (Fig 1) followed by mass spectrometry and matching the peptides to the protein sequence databases [29]. Recombinant constructs for the VSG LiTat1.3 and LiTat1.5 had been previously expressed in *Pichia pastoris* as a secreted product with evidence of post-translational processing [17]. rLiTat1.3 has a predicted molecular mass of 38 kDa from its amino acid sequence with 2 potential *N*-glycosylation sites. rLiTat1.5 is predicted to be 40.5 kDa with no potential *N*-glycosylation sites. The presence of two bands in the purified material for the rVSGs is likely to arise from either different post-translational modifications of the two species present or, possibly, be due to proteolysis as observed by Rogé *et al*. [17] when expressed in *Pichia pastoris*. All bands were positively identified as corresponding to the respective VSG by mass spectrometry but this data and not allow us to determine conclusively the difference between the two bands observed. rISG65 is predicted from its amino acid sequence to be 41.8 kDa with 2 potential *N*-glycosylation sites. Further analysis of the purified rISG65 by gel chromatography confirmed the presence of a monomeric molecule of 50–60 kDa as shown in Fig 2. Following PNGase F treatment to remove potential *N*-glycans, the apparent molecular mass of rISG65 was reduced, as determined using SDS-PAGE analysis, consistent with the presence of *N*-linked sugars (Fig 3).

### Diagnostic potential of the recombinant antigens for gambiense HAT and rhodesiense HAT

**Fig 4:**
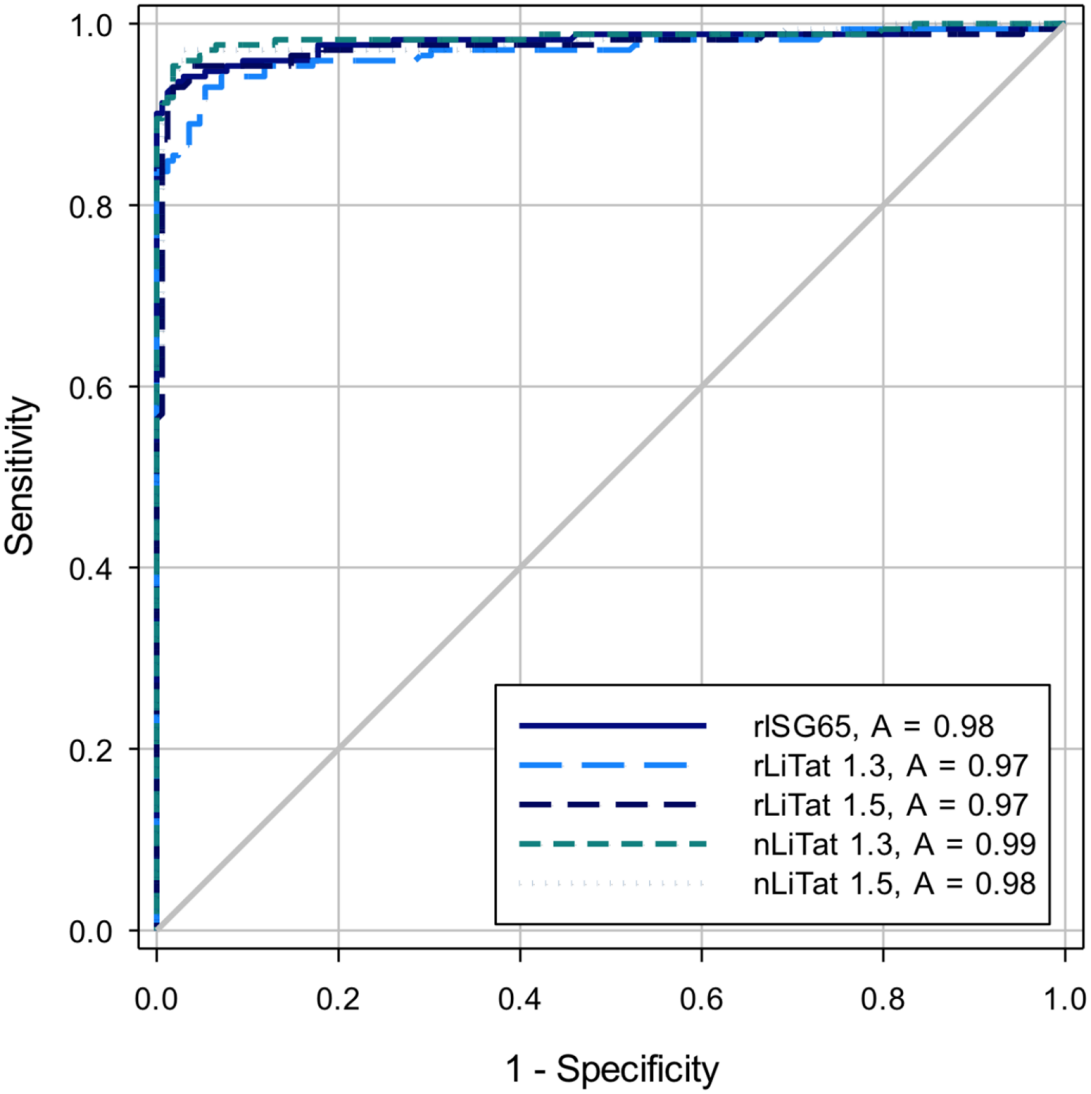
Receiver operator characteristic (ROC) curves and area under the curve (AUC) constructed from ELISA results obtained by testing sera from 172 *g-*HAT patients, 119 non-*g-*HAT controls and 50 non-*r-*HAT controls with nLiTat 1.3 (2 μg/ml), rLiTat 1.3 (4 μg/ml), nLiTat 1.5 (2 μg/ml), rLiTat 1.5 (4 μg/ml) and rISG65 (4 μg/ml).

The sera from 172 *g-*HAT patients, 119 non-*g-*HAT controls and 50 non-*r-*HAT controls were tested by ELISA with rISG65, rLiTat 1.3, rLiTat 1.5, nLiTat 1.3 and nLiTat 1.5. With the parasitological status as reference and the cut-off for each antigen set at its highest Youden index (sensitivity + specificity -100), each antigen displayed a sensitivity > 92.4% and a specificity > 94% (Table 2). The diagnostic potential of each antigen in function of varying OD_corr_ cut-off is represented in the Receiver Operating Curve (ROC) plots in Fig 4 showing areas under the curve (AUC) ranging from 0.97 to 0.98 for the recombinant antigens and from 0.98 to 0.99 for the native antigens (Fig 4 and Table 2). Pairwise comparison of the AUC obtained with the different antigens showed no statistically significant differences except between the AUC of rLiTat 1.3 and nLiTat 1.3 (Table 3).

**Table 2:** Area under the curve (AUC) and its 95% confidence interval (CI), Youden index, percent sensitivity (Se %), percent specificity (Sp %) and their respective 95% CI recorded for the different antigens when tested with sera from 172 *g-*HAT patients, 119 non-*g-* HAT controls and 50 non-*r-*HAT controls.

Antigen	AUC	95% CI	Youden	Se %	95% CI	Sp %	95% CI
rISG65	0.98	0.964–0.997	0.91	92.4	87.42%–95.91%	98.8	95.79%–99.86%
rLiTat 1.3	0.97	0.950–0.989	0.88	93.0	88.13%–96.34%	94.7	90.13%–97.54%
rLiTat 1.5	0.97	0.955–0.994	0.92	95.4	91.04%–97.97%	96.5	92.43%–98.69%
nLiTat 1.3	0.99	0.971–1.0	0.94	95.4	91.04%–97.97%	98.2	94.90%–99.63%
nLiTat 1.5	0.98	0.965–0.996	0.94	97.1	93.35%–99.05%	97.0	93.23%–99.03%

**Table 3:** Pairwise difference and *p* value of the Chi square between the areas under the curve recorded for the different antigens when tested with sera from 172 *T*.*b*. *g-*HAT patients, 119 non-*g-*HAT controls and 50 non-*r-*HAT controls. * = significantly different

	rLiTat 1.3	rLiTat 1.5	nLiTat 1.3	nLiTat 1.5
rISG65	0.011, *p* = 0.061	0.006, *p* = 0.291	0.005, *p* = 0.602	0.001, *p* = 0.981
rLiTat 1.3		0.005, *p* = 0.302	0.016, *p* = 0.040*	0.011, *p* = 0.188
rLiTat 1.5			0.011, *p* = 0.152	0.006, *p* = 0.423
nLiTat 1.3				0.005, *p* = 0.166

**Fig 5:**
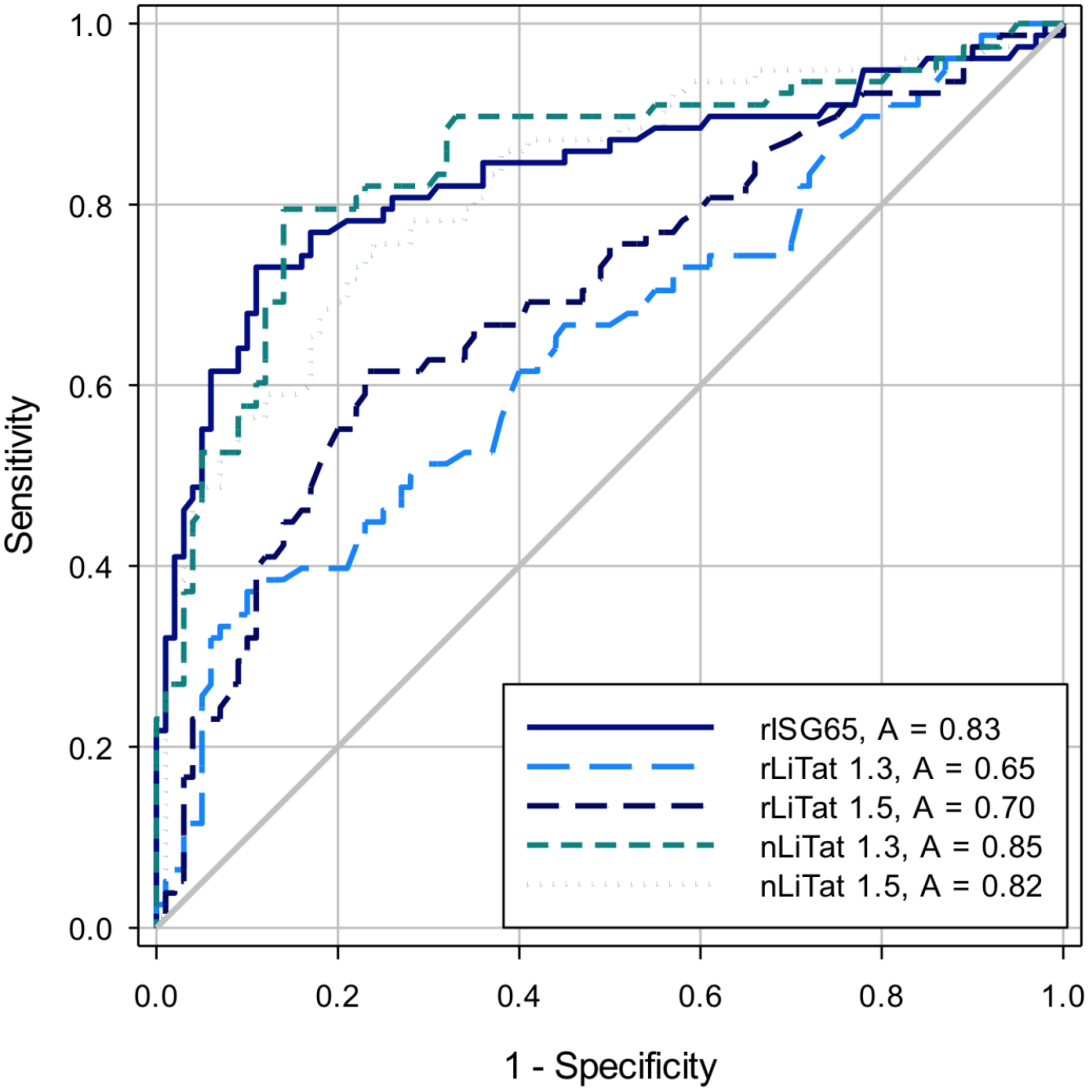
Receiver operator characteristic (ROC) curves and area under the curve (AUC) constructed from ELISA results obtained by testing sera from 78 *r-*HAT patients, 50 non-*r-*HAT controls and 50 non-*g-*HAT controls with nLiTat 1.3 (2 μg/ml), rLiTat 1.3 (4 μg/ml), nLiTat 1.5 (2 μg/ml), rLiTat 1.5 (4 μg/ml) and rISG65 (4 μg/ml).

In a similar way, the sera from 78 *r-*HAT patients, 50 non-*r-*HAT controls and 50 non-*g-*HAT controls were tested in ELISA with all the antigens. The results are represented in Fig 5 as ROC plots and in Table 4. Sensitivities and specificities ranged respectively from 37.2 to 79.5% and from 76 to 90% due to the poor reactivity of the antigens with *r-*sera. Significant differences in the AUC of the antigens were observed in more than half of the pairwise comparisons (Table 5).

**Table 4:** Area under the curve (AUC) and its 95% confidence interval (CI), Youden index, percent sensitivity (Se %), percent specificity (Sp %) and their respective 95% CI recorded for the different antigens when tested with sera from 78 *r-*HAT patients, 50 non-*r-*HAT controls and 50 non-*g-*HAT controls.

Antigen	AUC	95% CI	Youden	Se %	95% CI	Sp %	95% CI
rISG65	0.83	0.768–0.899	0.62	73.1	61.84%–82.50%	89.0	81.17%–94.38%
rLiTat 1.3	0.65	0.563–0.728	0.27	37.2	26.50%–48.87%	90.0	82.38%–95.10%
rLiTat 1.5	0.70	0.620–0.778	0.39	61.5	49.83%–72.34%	77.0	67.51%–84.83%
nLiTat 1.3	0.85	0.784–0.908	0.65	79.5	68.84%–87.80%	86.0	77.63%–92.13%
nLiTat 1.5	0.82	0.754–0.883	0.52	75.6	64.60%–84.65%	76.0	66.43%–83.98%

**Table 5:** Pairwise difference and *p* value of the Chi square between the areas under the curve recorded for the different antigens when tested with sera from 78 *r-*HAT patients, 50 non-*r-*HAT controls and 50 non-*g-*HAT controls. * = significantly different

	rLiTat 1.3	rLiTat 1.5	nLiTat 1.3	nLiTat 1.5
rISG65	0.188, *p* < 0.001*	0.135, *p* < 0.001*	0.012, *p* = 0.693	0.0143, *p* = 0.607
rLiTat 1.3		0.0531, *p* = 0.095	0.200, *p* < 0.001*	0.173, *p* < 0.001*
rLiTat 1.5			0.147, *p* < 0.001*	0.120, *p* = 0.001*
nLiTat 1.3				0.0265, *p* = 0.225

## Discussion

As the numbers of people infected with sleeping sickness continue to fall, the targeted elimination of the disease by 2030, as suggested by WHO, is conceivable [30]. However, many obstacles still need to be overcome including the development and availability of a reliable point of care test (POCT) and the development of oral delivery drugs which can be used in the remote areas where foci still exist. Such an assay will become even more essential for the monitoring and surveillance of remote areas in view of the targeted continuing decline of the prevalence. A recent examination of the currently available RDTs for serodiagnosis of *g-*HAT in West Africa demonstrated a lower specificity than expected (88%) but suggested a parallel use of both available tests could increase specificity and sensitivity [14]. These first generation RDTs contain native (n) nLiTat 1.3 and nLiTat 1.5 as antigens but research is ongoing to replace them by second and future generation RDTs containing recombinant antigens. A recombinant fragment of ISG65 has been expressed in *Escherichia coli* and used as an antigen in a prototype lateral flow device on its own [16] and in combination with native VSGs, VSG117 [15] or VSG 117 purified from *T*.*b*. *brucei* [31]. Although these tests showed a good sensitivity (88% to 98% for the single antigen device), the specificities only ranged between 65% and 93%. In the dual antigen prototypes, the specificities varied from 83% to 97% [15,16,30]. Recombinant antigens expressed in *E*. *coli* are not glycosylated and therefore may miss some critical epitopes with diagnostic potential that may be present on glycosylated and correctly folded native glycoproteins, such as ISG65. Correct glycosylation is often a requirement for proper folding of proteins and the absence of this in *E*.*coli* expression systems is likely to lead to both incorrect folding and absence of glycan containing epitopes. In contrast, eukaryotic expression systems, including insect and mammalian cells, yeast and protozoa, can yield glycosylated recombinants that can be engineered to be secreted into the culture medium [32]. The collection/harvesting of the medium can then be used as a first step in the purification process for such recombinant molecules.

In this study we describe the use of the *L*. *tarentolae* expression system (LEXSYS) to express *T b gambiense* surface antigens as secreted recombinant molecules. The native and recombinant VSG LiTat 1.5 showed comparable diagnostic accuracy when tested with *g-*HAT sera and non-HAT controls in the ELISA format as did the recombinant and native VSG LiTat 1.3. This suggests that recombinant VSG fragments could eventually replace the native forms currently used in RDT tests thus eliminating the complex and dangerous process of purifying native antigens from living, highly virulent infective parasites grown in laboratory rodents. Our results are similar to those reported for the expression of recombinant VSG LiTat 1.3 and VSG LiTat 1.5 fragments in *Pichia pastoris* [17]. Although VSGs LiTat 1.3 and LiTat 1.5 are *T*.*b*. *gambiense* specific and are not expressed in *T*.*b*. *rhodesiense* infections, both the recombinant and native forms of VSGs LiTat 1.3 and LiTat 1.5 reacted with a considerable number of sera from *r*-HAT patients yielding a sensitivity of up to 80% for native VSG LiTat 1.5. This could be due to epitopes on these VSG fragments that are not variant-specific but common to other variants, including variants expressed by other subspecies of *T*. *brucei* [18]. Among the recombinant antigens investigated here, it was rISG65 that showed the highest diagnostic potential for r-HAT with an AUC of 0.83. As ISG65 belongs to the invariant surface glycoprotein set of *T*. *brucei* the antigen should also be recognised by sera from *g*-HAT that do not contain antibodies against VSG LiTat 1.3 and LiTat 1.5. However, most sera from *g*-HAT patients used in the current study have been pre-selected by using CATT/*T*.*b*.*gambiense* as screening test during active case detection in the field. Therefore, a carefully designed prospective study with unbiased inclusion of participants is needed to confirm the hypothesis that rISG65 could detect patients that are not reactive with recombinant or native VSG LiTat 1.3 or VSG LiTat 1.5.

We conclude that rLiTat 1.3, rLiTat 1.5 and rISG65 can be expressed and post-translationally processed and secreted by *L*. *tarentolae* in a manner similar to the related kinetoplastid Trypanosome species. Moreover, the ease of engineering and production of recombinant proteins by this system allows for the development of tests for single or multiple diseases by combining different antigens on a single lateral flow test. We expect that rISG65, in combination with one or another recombinant VSG, will lead to the development of a powerful serodiagnostic test for *g*-HAT that will meet the ASSURED criteria for NTD diagnostics [11]. In addition *L tarentolae* has the potential to be used for the expression of antigens for other neglected tropical diseases caused by kinetidoplastid organisms as it appears to process the recombinant proteins in the same manner as the native antigens.
